# Authors' reply to: Interpretable multi-task prediction in rhabdomyolysis: promise and caveats

**DOI:** 10.1016/j.eclinm.2026.103897

**Published:** 2026-04-13

**Authors:** Chunli Liu, Jie Shi, Fengjuan Wang, Duo Li, Yu Luo, Bofan Yang, Yunlong Zhao, Li Zhang, Dingwei Yang, Heng Jin, Jie Song, Xiaoqin Guo, Haojun Fan, Qi Lv

**Affiliations:** a School of Disaster and Emergency Medicine, Tianjin University, Tianjin, China; b Department of Nephrology, National Key Laboratory of Kidney Diseases, National Clinical Research Center for Kidney Diseases, Military Logistics Research Key Laboratory of Field Disease Treatment, Beijing Key Laboratory of Kidney Disease Research, First Medical Center of Chinese PLA General Hospital, Beijing, China; c Department of Nephrology, Tianjin hospital of Tianjin University, Tianjin, China; d Department of Emergency Medicine, Tianjin Medical University General Hospital, Tianjin, China; e Characteristics Medical Center of Chinese People's Armed Police Force, Tianjin, China

We sincerely appreciate the thoughtful comments and constructive suggestions provided by the authors of the correspondence regarding our recently published study.^1^ We address the main points raised as follows:

## On the estimation of baseline serum creatinine (SCr)

We thank the authors for their attention to the estimation of baseline SCr. In our study, the diagnosis of acute kidney injury (AKI) strictly followed the KDIGO clinical practice guidelines, incorporating both serum creatinine changes and urine output criteria. In the MIMIC-IV and eICU-CRD databases, we used the official KDIGO-derived tables (e.g., kdigo_creatinine, kdigo_uo), which were generated based on validated KDIGO algorithms. Consequently, no recalculation of baseline SCr was needed, ensuring consistency and accuracy in AKI determination.

For the external validation cohort from China (BTMH), AKI diagnoses were based on explicit clinical documentation in the electronic medical record and further verified in collaboration with the clinical team. In the small subset of patients for whom pre-admission SCr was not available, we followed the approach described in the KDIGO 2012 guidelines by assuming an eGFR of 75 mL/min/1.73 m^2^ and back-calculating SCr using the MDRD equation. This method is considered reasonable when chronic kidney disease (CKD) is absent and is consistent with the recommendations by Bouchard et al. in Kidney International Reports (2021; https://doi.org/10.1016/j.ekir.2021.01.030),^2^ which support its use in populations with low CKD prevalence.

It is important to note that the proportion of missing SCr values in the training datasets was extremely low (0.6%). The estimated baseline SCr was used only to determine AKI labels in a very small number of patients within the external cohort and did not serve as an input variable for model training.^3^

We also clarified this methodology during peer review, and the approach was acknowledged by the reviewers.^4^

## On the definition of severe patients

We thank the authors for identifying this issue and have carefully reviewed the definition. In our study, severe patients were defined as those who developed both AKI and required RRT during hospitalization, those who experienced in-hospital mortality, or those with AKI classified as KDIGO stage 3. The published text inadvertently omitted the inclusion of KDIGO stage 3 patients.

The purpose of defining this severity category was to highlight a clinically meaningful subgroup with poor outcomes, thereby improving risk stratification and supporting early intervention strategies.

## On the handling of missing data

We appreciate the comments regarding missing data imputation. While we recognize that more sophisticated approaches, such as k-nearest neighbors or multiple imputation, may offer theoretical advantages, median imputation is widely used in clinical prediction studies based on electronic medical records (e.g., https://doi.org/10.1016/j.eclinm.2023.102409; https://doi.org/10.1249/MSS.0000000000002674).^5^, ^6^ Importantly, median imputation is straightforward, robust to outliers, computationally efficient, and facilitates practical model deployment.

In addition, the proportion of missing serum creatinine (SCr) values in the training datasets was very low (approximately 0.6%), which further supports the use of median imputation as a reasonable and pragmatic approach in this context.^7^

A key consideration for our study was the clinical applicability of the prediction system. Median imputation allows real-time completion of missing values in new patients without re-accessing the full training dataset, which is essential for implementing a timely and operational clinical decision-support tool.

## On model evaluation metrics

We agree that discrimination alone (AUC) does not fully reflect clinical utility. In addition to AUC,^8^ we reported a range of performance metrics—including sensitivity, specificity, accuracy, F1 score, PPV, and NPV—in the Supplementary Materials to provide a more comprehensive evaluation.

We appreciate the suggestion to include calibration curves, decision-curve analysis, and precision–recall curves. We are currently conducting a larger-scale follow-up study that will incorporate these additional evaluation measures, which we agree can further strengthen the clinical relevance of our model.^9^

We thank the authors of the correspondence and believe our clarifications will further enhance the understanding and transparency of our work.

## Contributors

CLL, JShi, and FJW contributed equally to this work. CLL, JS, FJW, and QL conceived and designed the study. DL, YL, BFY, YLZ, DWY, HJ, JSong, and LZ were responsible for data collection and curation. CLL and FJW conducted the data analysis. CLL, JShi, and FJW drafted the initial manuscript. XQG, HJF, and QL supervised the study and provided critical revisions. CLL, JShi, FJW, and QL had full access to and verified the data. All authors reviewed and approved the final version of the manuscript.

## Declaration of interests

The authors declare that they have no competing interests.
